# Iron and thiols as two major players in carcinogenesis: friends or foes?

**DOI:** 10.3389/fphar.2014.00200

**Published:** 2014-08-28

**Authors:** Shinya Toyokuni

**Affiliations:** Department of Pathology and Biological Responses, Nagoya University Graduate School of MedicineNagoya, Japan

**Keywords:** iron, carcinogenesis, glutathione, oxidative stress, cancer stem cell, ferroptosis, peroxiredoxins, Nrf2

## Abstract

Iron is the most abundant metal in the human body and mainly works as a cofactor for proteins such as hemoglobin and various enzymes. No independent life forms on earth can survive without iron. However, excess iron is intimately associated with carcinogenesis by increasing oxidative stress via its catalytic activity to generate hydroxyl radicals. Biomolecules with redox-active sulfhydryl function(s) (thiol compounds) are necessary for the maintenance of mildly reductive cellular environments to counteract oxidative stress, and for the execution of redox reactions for metabolism and detoxification. Involvement of glutathione *S*-transferase and thioredoxin has long attracted the attention of cancer researchers. Here, I update recent findings on the involvement of iron and thiol compounds during carcinogenesis and in cancer cells. It is now recognized that the cystine/glutamate transporter (antiporter) is intimately associated with ferroptosis, an iron-dependent, non-apoptotic form of cell death, observed in cancer cells, and also with cancer stem cells; the former with transporter blockage but the latter with its stabilization. Excess iron in the presence of oxygen appears the most common known mutagen. Ironically, the persistent activation of antioxidant systems via genetic alterations in *Nrf2* and *Keap1* also contributes to carcinogenesis. Therefore, it is difficult to conclude the role of iron and thiol compounds as friends or foes, which depends on the quantity/distribution and induction/flexibility, respectively. Avoiding further mutation would be the most helpful strategy for cancer prevention, and myriad of efforts are being made to sort out the weaknesses of cancer cells.

## Introduction

**Figure 1:**
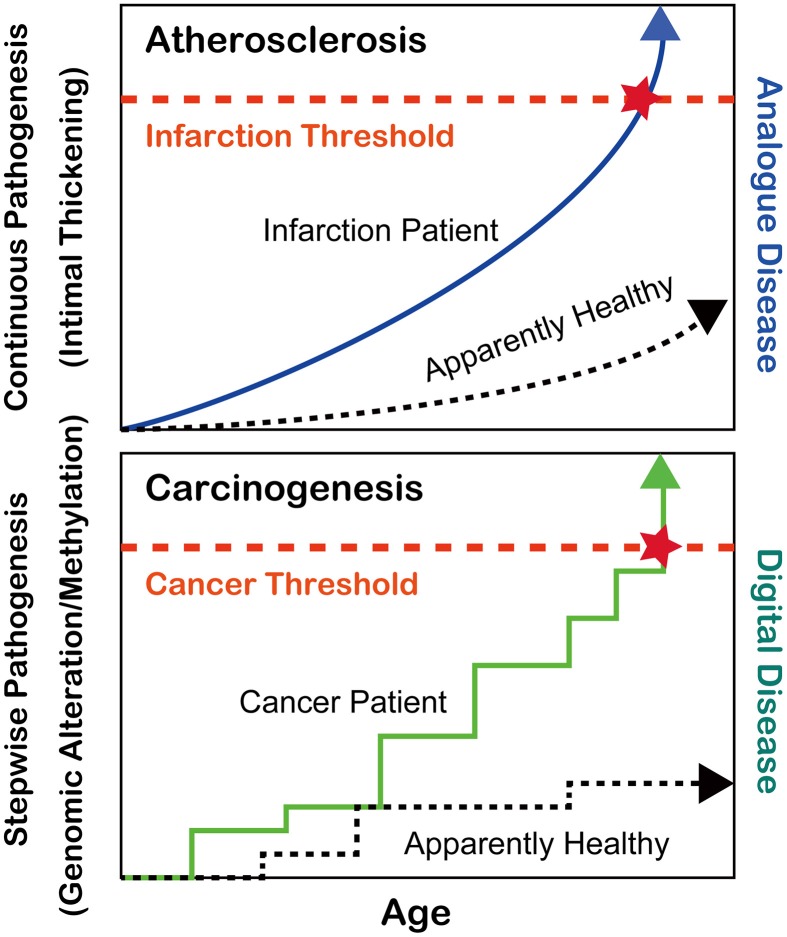
**Differences between atherosclerosis and carcinogenesis as oxidative stress-induced diseases**.

During the past 6–7 decades following World War II, the average human lifespan has been enormously extended from less than 50 years to nearly or more than 80 years in most developed countries (https://www.cia.gov/library/publications/the-world-factbook/rankorder/2102rank.html). This has been achieved at least in part by the discovery of antibiotics against bacterial infections such as tuberculosis, which had been continuously present deadly diseases until that period (Zhang, 2005). After the major human conquest over bacterial diseases, two pathologic conditions, atherosclerosis and cancer, have become the most common causes of human mortality. Atherosclerosis via the thickening of the arterial intima and rupture of atheromatous plaques causes myocardial and cerebral infarctions, which either kill the patients or dramatically decrease their quality of lives (Beckman et al., 2002). Atherosclerosis is, in a sense, an “analog” disease in that apparently nobody can escape from it (Kumar et al., 2013); rather, the speed of disease progression is much different among individuals, which is revealed with mathematical models (Hao and Friedman, 2014) (Figure 1). In contrast, cancer is a “digital” disease, which means that it consists of stepwise processes and is all or nothing for the generation of a malignant tumor (Weinberg, 2013) (Figure 1). Some patients develop secondary or even tertiary cancers after successful cancer treatments (Travis et al., 2013). In this review, I will focus on the recent advancements in the understanding of cancer regarding iron and thiol compounds that appears independent but are interdependent in many aspects.

## Cancer as a genomic disease

**Figure 2:**
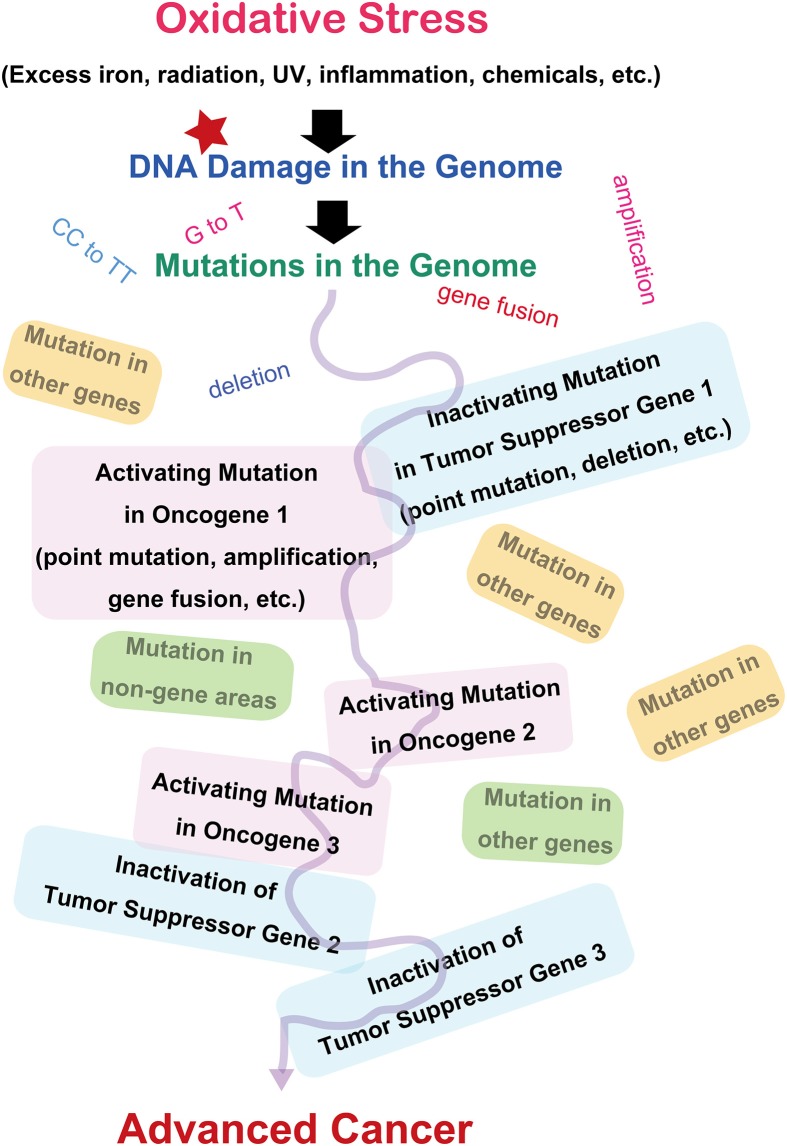
**Molecular carcinogenic processes in terms of oxidative stress**.

Since the discovery of oncogenes, decades of studies have revealed that cancer is basically a disease of the genomic alteration (Weinberg, 2013). Alteration of genome information after genomic damage and its inadequate repair is responsible for cancer development, and the alterations should occur in specific genes designated as oncogenes or tumor suppressor genes (Figure 2). There are more than 100 oncogenes identified thus far, and all of these genes are associated with cellular proliferation (Weinberg, 2013). Oncogene activation is the result of specific mutations in genes, leading to persistent activation of cellular signals toward proliferation. The genetic alterations include point mutations, gene amplifications, or gene fusions (Stratton et al., 2009; Pleasance et al., 2010). Tumor suppressor genes work as guardians of the genome by arresting the cell cycle, repairing the genome, and even inducing apoptosis after unrepairable excessive genomic injury occurs. These genes are inactivated during the carcinogenic process. It is now recognized that several (e.g., 5–8) independent or interdependent events of mixed activation of oncogenes and inactivation of tumor suppressor genes are necessary to generate a malignant tumor (Hanahan and Weinberg, 2000). Certain genetic alterations work as an instigator, facilitating the occurrence of sequential independent alterations at a significantly higher incidence (mutator phenotype) (Loeb, 2001). This is especially true of repair genes for the genome. Both alleles have to be disrupted to inactivate tumor suppressor genes. In addition, not only genetic alterations but also epigenetic changes (methylation of the promoter region) are important in halting the expression of specific genes (Feinberg et al., 2006).

As an exception, epigenetic alterations may lead to a malignant tumor in childhood cancer (Esteller and Herman, 2002). Recently, it was shown that doxycycline-controlled reprogrammable transgenic mice overexpressing the four Yamanaka factors (*Oct3/4*, *Sox2*, *Klf4*, and *c-Myc*) for induced pluripotent stem cell generation develop several cancers similar to childhood blastoma-type cancer that do not revert to normal and continue dysplastic growth, even after switching off those genes. Surprisingly, these cancers reportedly do not have major alterations of the genes, suggesting the importance of epigenetic mechanisms as well (Ohnishi et al., 2014). However, it was recently determined by next generation sequencing that genes regulating epigenetic mechanisms are one of the major targets of carcinogenesis in certain cancers such as leukemia and breast carcinoma (Smith et al., 2010; Dawson and Kouzarides, 2012). Thus, in most cases, genetic alterations regulate epigenetic mechanisms.

## Iron as a risk of cancer

**Figure 3:**
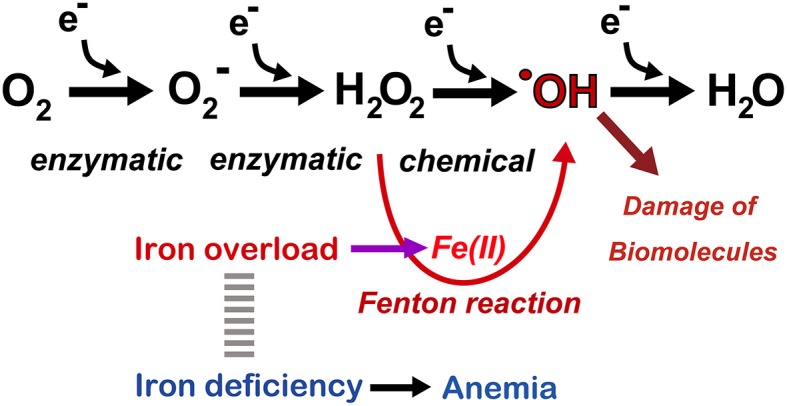
**Oxygen as a medium for electron flow and the associated role of catalytic ferrous iron (Fe[II]) toward Fenton reaction**. Note that only a small fraction generates hydroxyl radicals (^·^OH). Refer to text and **Figure 7**.

Iron is the most abundant metal in the human body. Approximately 4 g is present in normal adult humans. Thus, far, no life on earth can live without iron. Simultaneously, most of higher organisms, including humans, cannot survive without oxygen for 5 min. Oxygen is transported throughout the body by the heme moiety of hemoglobin, which contains as much as ~60% of the total iron in the body (Wriggleworth and Baum, 1980; Toyokuni, 2011a). Thus, there is a natural affinity between iron and oxygen. The most important characteristic of molecular oxygen is that it is easy to be reduced via one or more of four one-electron transfer processes, ending with the formation of water (Figure 3). During this process, superoxide, hydrogen peroxide, and hydroxyl radicals may be generated as intermediates either via enzymatic or chemical reactions. Hydroxyl radicals are most reactive among chemical species of the biological system. Fortunately, unfavorable reactions of this kind is usually prevented via antioxidative mechanisms (Halliwell and Gutteridge, 2007; Toyokuni, 2011b).

Whereas iron is an essential component, stable Fe[III] is hardly soluble at neutral pH (10^−17^ M) (Lippard and Berg, 1994). Therefore, precise and overlapping regulatory mechanisms exist in iron metabolism, and thus, only subtle amounts of catalytic iron are present in normal physiological conditions. Pathologic conditions, such as failure of iron regulatory sensors, repeated hemorrhage and chronic inflammation concomitant with continued parenchymal cell death, result in iron overload in the corresponding locations (Ganz, 2003), leading to oxidative damage through the Fenton reaction (Minotti and Aust, 1989; Miller et al., 1990; Halliwell and Gutteridge, 2007). Hydroxyl radicals are the most reactive species in the biological system, and the Fenton reaction *in vivo* appears to occur in the presence of catalytic ferrous iron (Samuni et al., 1983; Toyokuni, 2009), leading to the extensive scission, modification (Dizdaroglu, 1991) and cross-linking of biomolecules (Dizdaroglu, 1991). Such oxidative molecules eventually induce genomic alterations, increasing the risk of carcinogenesis. Recently, prompt formation of mono- or poly-iron Fe^IV^ = O (ferryl) species was suggested at the aqueous interface (Enami et al., 2014).

Indeed, iron overload has been associated with carcinogenesis both in human and animal experiments. It is well known that there is no active excretion pathway for iron except for hemorrhage, presumably due to the supreme biological importance of iron. Intriguingly, a US epidemiological study published in 2008 reported that for peripheral arterial disease patients, phlebotomy twice a year reduced the incidence of visceral cancer by 35% and the cancer mortality by 61% in a randomized trial involving 1277 patients. In this study, iron reduction did not stop the progression of atherosclerosis, as originally intended, but did unexpectedly prevent carcinogenesis, including that of most common cancers (lung, colon, prostate, etc.) (Zacharski et al., 2007).

**Figure 4:**
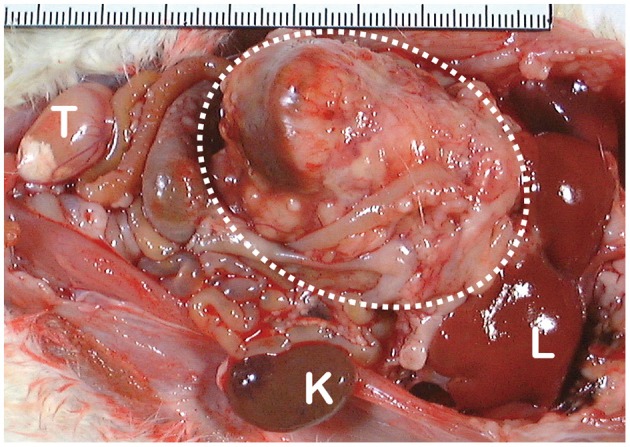
**Macroscopic appearance of ferric nitrilotriacetate (Fe-NTA)-induced renal cell carcinoma (interrupted circle; tumor diameter is more than 40 mm)**. K, normal kidney of the opposite side; L, liver; T, testis.

There are several review articles published on the direct demonstration of iron overload and carcinogenesis in animal experiments (Toyokuni, 1996, 2002, 2009; Beguin et al., 2014), which I summarize as Table 1. Now, the role of iron in human carcinogenesis remains under intensive discussion (Cho et al., 2013; Fonseca-Nunes et al., 2014). Here, I discuss the recent results from a genuine Fenton reaction-induced carcinogenesis model generated by intraperitoneal injections of an iron chelate, ferric nitrilotriacetate (Fe-NTA), to rodents (Ebina et al., 1986; Li et al., 1987). Nitrilotriacetate (NTA) has been used as a component of detergents in Canada because of its potent chelating activity with a variety of metals, including iron (Anderson et al., 1982). First, Fe-NTA was used to load Fe[III] to transferrin, a serum iron transporting protein, in biochemistry laboratories (Pootrakul et al., 1977). Then, it was used by Awai et al. to generate an animal model of hemochromatosis (Awai et al., 1979). Of note, Okada and Midorikawa found that Fe-NTA induces renal cell carcinoma (RCC) after repeated intraperitoneal administration in wild-type rats (Figure 4) that were accidentally under observation for more than 1 year after the confirmation of iron accumulation in the liver (Okada and Midorikawa, 1982). Fe-NTA is soluble at neutral pH and is the most potent catalyst thus far of Fenton reaction with 3–4 free iron ligands (Toyokuni and Sagripanti, 1992, 1993).

**Table 1:** **Models of iron-induced carcinogenesis using wild-type animals**.

**Iron compounds**	**Administration route**	**Species**	**Induced cancer**	**References**
Iron oxide	Inhalation	Mouse	Lung adenocarcinoma, fibrosarcoma	Campbell, 1940
Iron dextran complex	Intramuscular	Rat	Spindle cell sarcoma	Richmond, 1959
Ferric nitrilotriacetate	Intraperitoneal	Rat	Renal cell carcinoma	Ebina et al., 1986; Nishiyama et al., 1995; Tanaka et al., 2000, 2004; Akatsuka et al., 2012
Ferric nitrilotriacetate	Intraperitoneal	Mouse	Renal cell carcinoma	Li et al., 1987
Ferric saccharate	Intraperitoneal	Rat	Malignant mesothelioma	Okada et al., 1989; Hu et al., 2010
Ferric ethylene-diamine- N,N′-diacetate	Intraperitoneal	Rat	Renal cell carcinoma	Liu and Okada, 1994

The models shown above demonstrate the carcinogenicity of iron compounds in rodents.

**Figure 5:**
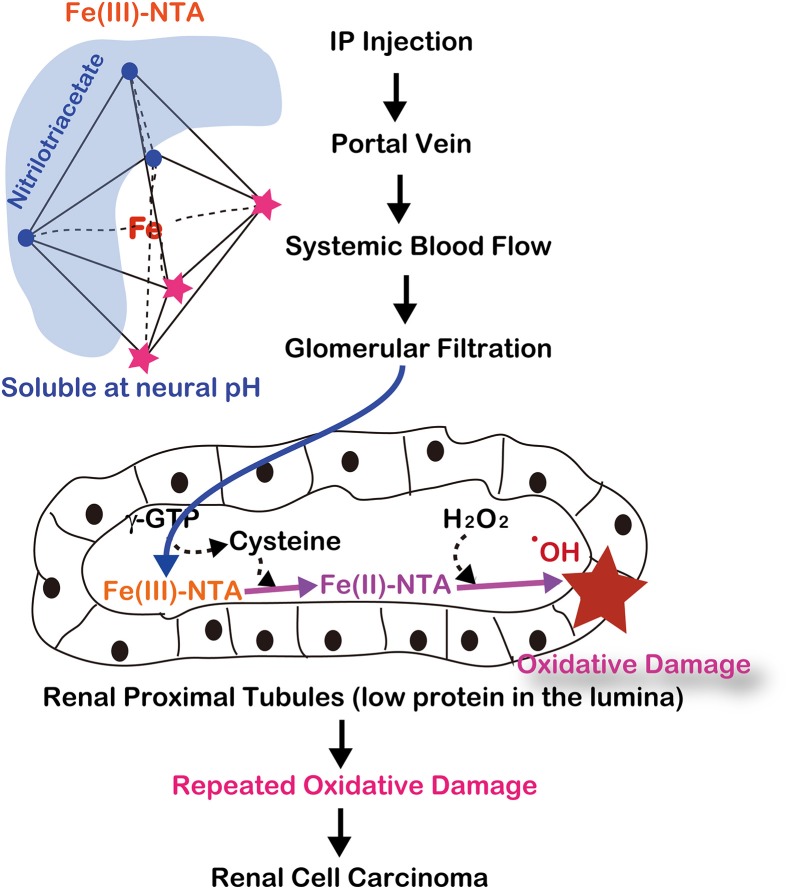
**Molecular mechanism of ferric nitrilotriacetate (Fe-NTA)-induced oxidative damage of renal proximal tubules after a single intraperitoneal injection**. This depends on two distinct characteristics of renal proximal tubular lumina: paucity of antioxidattive proteins such as albumin and reductive environment through the presence of L-cysteine with GSH cycles.

After intraperitoneal injection, Fe-NTA is absorbed through the peritoneum into the portal vein and then enters the systemic blood flow. Thereafter, Fe-NTA is filtered through the glomeruli into the lumina of the renal proximal tubules, where Fe(III)-NTA is reduced to Fe(II)-NTA, presumably by the presence of L-cysteine (Okada et al., 1993; Okada, 1996) (Figure 5). The Fenton reaction indeed occurs *in vivo* in rats and mice because a variety of modified products are demonstrated in this model, including 4-hydroxy-2-nonenal (HNE) (Toyokuni et al., 1997a), HNE-modified proteins (Toyokuni et al., 1994b; Fukuda et al., 1996b), other lipid peroxidation products (Toyokuni et al., 1990; Uchida et al., 1995), 8-oxoguanine (Toyokuni et al., 1994a, 1997b), thymine-tyrosine cross-links (Toyokuni et al., 1995a) and other oxidative DNA base modifications (Toyokuni et al., 1994a). DNA single- and double-stranded breaks have also been shown *in vitro* (Toyokuni and Sagripanti, 1993, 1996).

Recently, we demonstrated an abundance of Fe(II) in the lumina of renal proximal tubules after Fe-NTA injection (Mukaide et al., 2014) with a novel fluorescent probe (RhoNox-1) specific for catalytic ferrous iron (Hirayama et al., 2013). Furthermore, we showed that the induced RCCs have massive chromosomal alterations similar to those of human cancers, of which the amplification of *c-Met* (receptor for hepatocyte growth factor) and deletion of *Cdkn2a/2b* (*p16*^*Ink4a*^/*p15*^*Ink4b*^ tumor suppressor genes) are most common (Tanaka et al., 1999; Akatsuka et al., 2012). The latter occurs early in carcinogenesis (Hiroyasu et al., 2002). Considering that it is rare thus far to find massive chromosomal alterations in any other carcinogenesis model using wild-type animals, we believe that iron overload is one of the most important risk factors in human carcinogenesis as well. Of note, asbestos- (Jiang et al., 2012) and multi-walled carbon nanotube-induced (Nagai et al., 2011) mesothelial carcinogenesis in wild-type rats are the models that confer massive chromosomal alterations including homozygous deletion of *Cdkn2a/2b*. We believe that these are through local iron overload (Toyokuni, 2013b).

There are several distinct human diseases that preclude cancer via iron overload (Table 2). Endometriosis is defined by the presence of endometrial tissue outside of the uterine cavity and occurs in as many as 10% of women in their reproductive years. An epidemiological study revealed that ovarian endometriosis is associated with a high risk for clear cell carcinoma (Pearce et al., 2012). Because monthly menstrual hemorrhage occurs in these ectopic tissues, local iron overload is generated *in situ* (Yamaguchi et al., 2008). We recently studied endometriosis-associated ovarian clear cell carcinoma with an array-based comparative genome hybridization and found that *c-Met* (the same target gene as those of Fe-NTA-induced RCCs) is the most frequently amplified gene (Yamashita et al., 2013). Recently, it was shown with the use of knockout mice for *Cdkn2a/2b* and/or *Pten* that DNA double-stranded breaks cooperate with the loss of Ink4 and Arf (protein products from *Cdkn2a/2b* after alternative splicing) tumor suppressors to generate glioblastomas with frequent *c-Met* amplification (Camacho et al., 2014). It is remarkable that all of the necessary genomic alterations occurred in the Fe-NTA-induced RCC model of wild-type animals.

**Table 2:** **Human carcinogenesis associated with iron overload**.

	**Target organ(s)**	**Pathogenesis**	**Cancer**	**Major genetic alterations**	**References**
Genetic hemochromatosis	Liver	Hereditary disorder (types 1–5); excessive iron absorption	Hepatocellular carcinoma, gastric cancer, etc.		Fracanzani et al., 2001; Agudo et al., 2013
Viral hepatitis B and C	Liver	Autoimmunity-induced hepatocyte damage and iron accumulation	Hepatocellular carcinoma		Bonkovsky et al., 1997; Kato et al., 2007
Endometriosis	Ovary	Monthly menstrual hemorrhage in ectopic endometrial tissue	Clear cell carcinoma, endometrioid adenocarcinoma	Amp of *c-MET*	Pearce et al., 2012; Yamashita et al., 2013
Asbestos exposure	Mesothelium, lung	Content and adsorption of asbestos fiber; chronic inflammation by foreign body	Malignant mesothelioma; lung cancer	HD of *CDKN2A/2B*, Amp of *c-MET*	Jiang et al., 2012; Aierken et al., 2014

Amp, amplification; HD, homozygous deletion.

**Figure 6:**
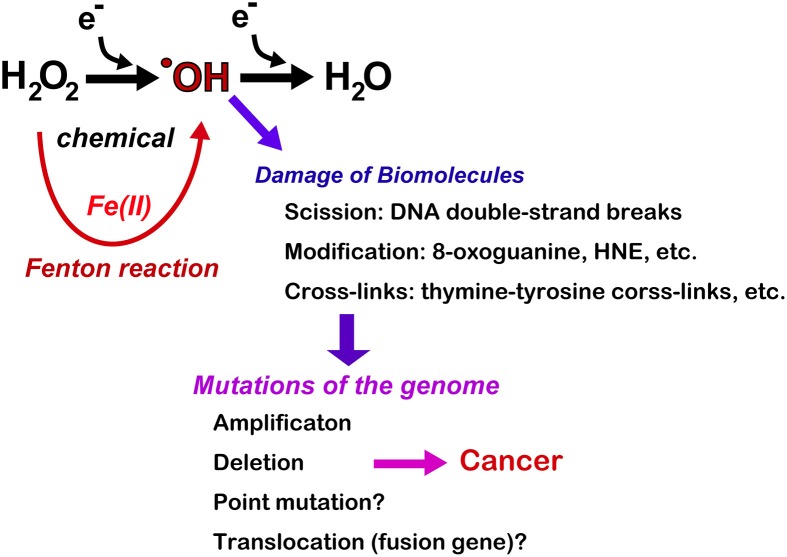
**Consequences of Fenton reaction in the genome DNA during carcinogenesis**. HNE, 4-hydroxy-2-nonenal.

Together, these results indicate that the Fenton reaction can induce deletion/amplification mutations in target genes during carcinogenesis, presumably through DNA double-stranded breaks (Figure 6). Currently, next-generation sequencing studies are in progress for the above-mentioned tumors and may help determine whether iron overload can induce driver point mutations and create fusion genes through chromosomal translocations.

## Regulation of hydrogen peroxide via thiol compounds

Here I discuss the other partner of Fenton reaction, hydrogen peroxide. Hydrogen peroxide, a non-radical species, is a normal metabolite occurring at an approximately 10 nM intracellular concentration. Its increase in concentration may initiate Fenton reaction in the presence of catalytic Fe[II]. In the liver, which has one of the highest metabolic activities in the body, hydrogen peroxide is produced at 50 nmol/min/g tissue (Sies, 2014). In the cells and in tissues with iron overload, the regulation of the concentration and localization of hydrogen peroxide is a critical issue. It is now widely accepted that hydrogen peroxide is utilized in metabolic regulation in ways similar to diffusible gasses such as CO, NO, and H_2_S (Yang et al., 2008).

A major source of hydrogen peroxide comes from the dismutation of the superoxide anion radical, which is mainly generated through NAD(P)H oxidases operated under the control of growth factors and cytokines, such as interleukin-1 and tumor necrosis factor-α (Jiang et al., 2011). This mechanism is actively used for antibacterial defense in neutrophils and macrophages during inflammation (Bedard and Krause, 2007). Another major source of hydrogen peroxide resides in the physiological mitochondrial processes through Complex I, II, and III (Cadenas and Davies, 2000).

**Figure 7:**
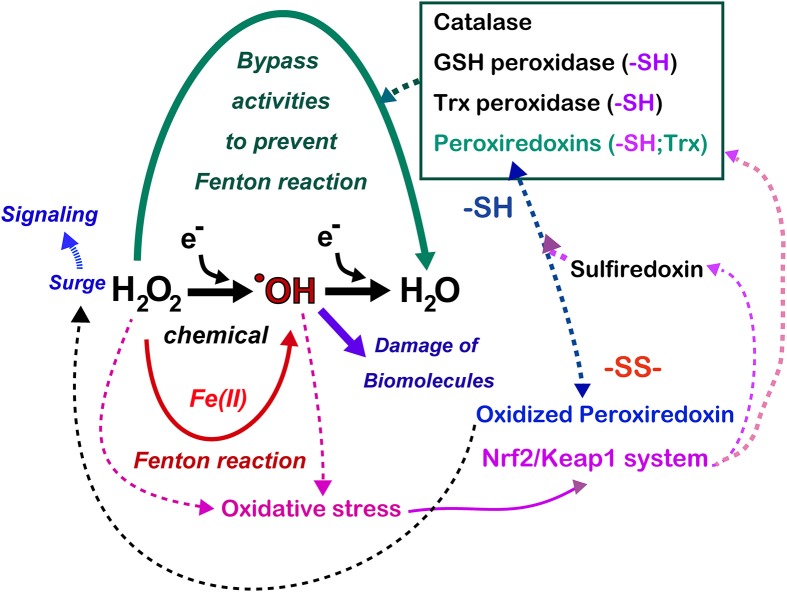
**Antagonizing role of iron and thiol compounds**. Numerous overlapping mechanisms using thiol compounds exist to decompose hydrogen peroxide to water in order to bypass the generation of hydroxyl radicals. Hydrogen peroxide is now recognized as a signaling molecule whose main regulator is the peroxiredoxin/sulfiredoxin systems, which are at least partially under the control of the Nrf2/Keap1 system. Some cancers hijack Nrf2/Keap1 system with mutation in these genes, which persistently activate antioxidant systems in the cancer cells. Refer to text and Figure 8 for details.

The metabolic elimination of hydrogen peroxide includes the catalytic reaction, which is performed by catalase in peroxisomes as well as by numerous peroxidases (Figure 7). In addition, in tissues, hydrogen peroxide diffuses away from its source across the plasma membrane to the extracellular space, or even to adjoining cells, occurs (Giorgio et al., 2007). Various peroxidases are under the control of metabolic signals, and the most potent peroxidase is peroxiredoxins (Rhee et al., 2005). The 10^6^-fold higher rate constant of the reaction of hydrogen peroxide with the cysteine thiolate in peroxiredoxins using thioredoxin as a substrate in comparison to most other deprotonated thiol compounds gives them a major role in the biological chemistry of hydrogen peroxide removal (Winterbourn, 2013). However, cysteine residues of peroxiredoxins are easily hyperoxidized to cysteine sulfinic acid, resulting in the inactivation of peroxidase activity. As a result, if this occurs, there is an accumulation of hydrogen peroxide, allowing the oxidation of specific target proteins, a phenomenon that is comparable to the opening of a gate for signaling (Wood et al., 2003). This is the molecular basis for hydrogen peroxide compartmentation in signaling (Antunes and Cadenas, 2000) (Figure 7). This activation is finally shut down by sulfiredoxin, which recovers hyperoxidized peroxiredoxins (Jeong et al., 2012). Glutathione peroxidases in various distinct subcellular compartments and cells play a major function in the regulation of hydrogen peroxide and lipid peroxides (Brigelius-Flohe and Maiorino, 2013). Glutathione reductase and the efflux of oxidized glutathione (GSSG) allow the maintenance of the fraction of reduced glutathione (Sies, 1999). Thioredoxin peroxidase, a selenium-dependent enzyme, also has an important role in the elimination of hydrogen peroxide (Lu and Holmgren, 2014).

Recently, hydrogen peroxide has been shown to use water channels, the aquaporins, to cross the lipid membrane more rapidly than diffusion allows (Bienert et al., 2006). Specific aquaporins promote the diffusion of hydrogen peroxide and are thus referred to as peroxiporins (Bienert et al., 2007). Aquaporin-8 can modulate the transport of hydrogen peroxide produced by NAD(P)H oxidase in leukemia cells (Bienert et al., 2007), suggesting novel targets for cancer therapy in that, in general, cancer cells are under persistent oxidative stress (Toyokuni et al., 1995b).

## Thiol-dependent antioxidant systems and cancer

The GSH and thioredoxin systems are generally activated in cancer (Tanaka et al., 1997; Dutta et al., 2005; Nogueira and Hay, 2013; Penney and Roy, 2013; Traverso et al., 2013). Among them, there has been much interest in the overexpression of GSH *S*-transferase in rodent hepatocarcinogenesis (Hatayama et al., 1993) and in human cancers (Huang et al., 2013; Tang et al., 2013). GSH *S*-transferase pi has now been connected with peroxiredoxin-6 for its recovery of peroxidase activity (Zhou et al., 2013). Acute temporary as well as persistent overexpression of GSH *S*-transferase pi was observed during renal carcinogenesis induced by Fe-NTA (Fukuda et al., 1996a; Tanaka et al., 1998).

**Figure 8:**
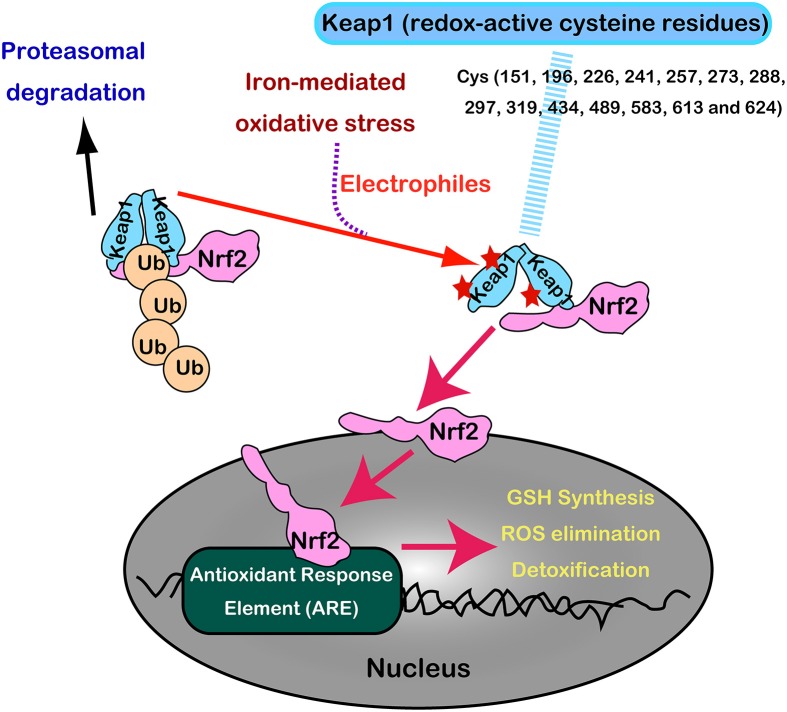
**Regulation of Nrf2 transcription machinery through oxidative stress sensor, Keap1, with numerous redox-reactive cysteine residues**.

Nrf2 and Keap1 are now recognized as a master regulatory transcription system for antioxidant enzymes (GSH synthesis, hydrogen peroxide removal, detoxification, drug excretion, and NADPH synthesis) (Suzuki et al., 2013). Under normal conditions, Nrf2 is constitutively produced but is inactivated in the cytoplasm following its interaction with Keap1 by ubiquitination and proteasomal degradation. Keap1 is indeed a sensor molecule for oxidative stress. The multiple cysteine residues on Keap1, which are ultrasensitive to electrophiles, are critically important for the binding with Nrf2 (Itoh et al., 1997, 1999; Mitsuishi et al., 2012) (Figure 8).

With the aid of next generation sequencing, it has become clear that Nrf2 is consistently activated in certain cancers with various mutations. Mutually exclusive Nrf2 mutations or Keap1 mutations are observed in cancers for Nrf2 to be localized in the nucleus (Mitsuishi et al., 2012). Sulfiredoxin is also under the transcriptional regulation of Nrf2 (Jeong et al., 2012). Thus, cancer cells have hijacked this system, making them consistently more resistant to oxidative stress (Figure 7). Recently, peroxiredoxin 1, 4, and 6 were shown either to enhance tumor progression or to promote metastasis (Ishii et al., 2012).

## Cancer stem cells and thiol metabolism

Stem cells are defined as undifferentiated immature cells that, upon certain stimuli or signaling, differentiate into a planned type of mature cell(s). Cancer stem cells represent a distinct subset, namely, cells that have acquired all the necessary genetic and epigenetic alterations but are usually quiescent and divide only if necessary (Holland et al., 2013). This feature is in contrast to the cancer tissue as a whole, which is exposed to persistent oxidative stress (Toyokuni et al., 1995b). In this sense, cancer stem cells constitute small heavenly territories in cancer tissue. In transplant experiments, theoretically, even a single cancer stem cell can generate a large tumor. Thus, the existence of cancer stem cells has been used to explain chemotherapy-resistance in such a dormant state. These cancer stem cells are more resistant than non-stem cancer cells to cytotoxic chemicals.

**Figure 9:**
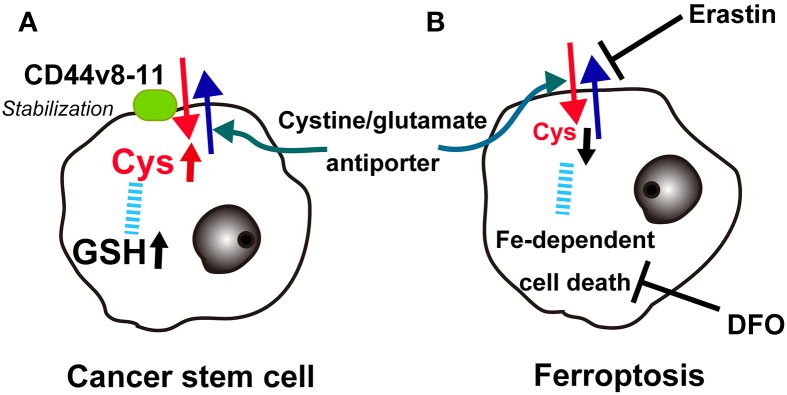
**Key role of the cystine/glutamate antiporter in cancer cells**. **(A)** Overexpression of CD44v(8-11) stabilizes the cystine/glutamate antiporter to increase cysteine and glutathione (GSH) in cancer stem cells. **(B)** Conversely, erastin blocks the cystine/glutamate antiporter, lowers cysteine and finally induces iron-dependent cancer cell death (ferroptosis), which can be blocked with deferoxamine (DFO).

Recently, Hideyuki Saya's group reported that cancer stem cells in certain cancers present CD44 variant 8-10 (CD44v) isoform on the plasma membrane, which stabilizes the cystine/glutamate transporter (antiporter; xCT), leading to increased GSH (Ishimoto et al., 2011). This may at least partially explain the robustness of cancer cell defenses against oxidative stress, including those involved in chemotherapy-resistance. There is already an xCT antagonist, sulfasalazine, and clinical trials are underway in advanced gastric cancer in Japan (Figure 9A).

## Ferroptosis

Recently, a different type of cell death other than necrosis, apoptosis, or autophagy was reported in cancer cells. The oncogenic RAS-selective lethal small molecule erastin triggers a unique iron-dependent form of non-apoptotic cell death called ferroptosis (Dixon et al., 2012). Similar to glutamate, erastin inhibits cystine uptake by the cystine/glutamate antiporter, inducing a void of antioxidant defenses, resulting in iron-dependent oxidative stress. Interestingly, this type of cell death is inhibited by an iron chelator, deferoxamine, which removes cellular iron (Figure 9B). Deferoxamine functions in clear contrast to NTA in that it blocks all 6 ligands of iron (Toyokuni and Sagripanti, 1992). The authors suggest that one of more yet unidentified iron-dependent enzymes are functioning as a core lethal mechanism for ferroptosis (Dixon et al., 2012).

## Future cancer prevention and cancer therapeutics

It is generally accepted that chronic oxidative stress via excess iron leads to carcinogenesis, presumably through hydroxyl radicals, and that most of the defense system is associated with thiol compounds whereas iron and thiol compounds are apparently essential elements. These two are antagonistic in abundance, and their characteristics are distinct (Table 3).

**Table 3:** **Antagonizing roles of iron and thiols**.

	**Iron**	**Thiol compounds**
Transition	Fe(II), transport across membrane, cytosol/Fe(III), extracellular)	-SH(reduced)/-SS-(oxidized)
Reaction	Catalytic Fe(II): Fenton reaction in the presence of hydrogen peroxide; oxidative damage by hydroxyl radicals, irreversible but usually limited with various preventive mechanisms	Common; non-destructive; redox regulation; usually reversible with reducing enzymes (Figure 7); free form, H_2_S, SH^−^ (Nishida et al., 2012)
Biological significance	Cofactor of proteins (heme)/enzymes (catalytic site); stored as ferritin; oxygen transport and storage	Formation of mildly reductive intracellular environments; mostly present as cysteine residue in peptides (GSH) or proteins (thioredoxin, metallothionein, etc.); cystine when oxidized; redox signal
Metabolism	Slow; essential nutrient; nearly closed system (whole 4 g in adults; 1 mg in and out daily); no excretion pathway except bleeding (hemoglobin); transported by transferrin and its receptor system	Fast; cysteine is synthesized from methionine (essential amino acid)
Regulation	Iron transporters; HFE, hepcidin, IRP-1 and -2 (posttranscriptional) (Hentze and Kuhn, 1996), etc.	Transcription factors: Nrf2/Keap1, AP-1, NF-κB (Schenk et al., 1994; Jeong et al., 2012), etc.
Deficiency/excess/toxicity	Deficiency causes anemia; excess leads to oxidative tissue damage and sometimes carcinogenesis	Toxicity known for certain thiol compounds to initiate Fenton-like reaction (Munday, 1989); persistent activation of the associated systems is observed in cancer and its stem cell (refer to text for details)
Molecular affinity	O_2_, CO, NO; transferrin, siderophore (Devireddy et al., 2010); mitoferrin (Shaw et al., 2006), frataxin (Schmucker et al., 2011), Fe-S cluster (Hentze et al., 2010)	Electrophiles (Dennehy et al., 2006), toxic metals (Hg, Cd, Pb, and As) (Quig, 1998), 8-nitro-cGMP (Nishida et al., 2012)

For cancer prevention, we recognize that cancers of different tissues are completely different diseases. Specific risks are present for each type of cancer, and we have to decrease specific risks as early as possible in our lives. For example, asbestos is a definite carcinogen, causing malignant mesothelioma, and smoking increases the risk of more than 20 different cancers, including laryngeal and lung cancers (Toyokuni, 2013a). Today we demand practical methods for prevention of overall cancer. Antioxidant systems, if deficient, have to be supplemented. However, thus far, antioxidant supplementation has not officially been recommended based on the results of epidemiological studies (Bjelakovic et al., 2004, 2007). Excessive supplementation with β-carotene even increased the risk of lung cancer in smokers (Albanes et al., 1996). Here appropriate iron reduction via blood donation or phlebotomy may be a potential method of cancer prevention. Among the three components of iron, oxygen, and thiol compounds, we can modify only iron status after all. Humans live much longer than they did 70 years ago, following the conquest of major infectious diseases. After reaching middle age, iron is found in excess, especially in men of well-developed countries, because there is no other way to excrete iron (Toyokuni, 2011a). This theory requires further epidemiological studies and clinical trials for demonstration. Reportedly, cancer may hijack cytokine systems (e.g., SMAD4) via mutation to collect iron for proliferation (Wang et al., 2005). Notably, there is an opposing report that iron deficiency accelerates Helicobacter pylori-induced carcinogenesis in rodents and humans (Noto et al., 2013). Iron deficiency appears to enhance the virulence of Helicobacter pylori, which definitely requires iron to live. This fact needs to be further discussed. Iron deficiency should be avoided because it causes anemia (hemoglobin) and muscle weakness (myoglobin).

Currently, antibody therapies (Scott et al., 2012) and small-molecular-weight kinase inhibitors (Fabbro et al., 2012) are popular and work well as individualized therapies for specific cancers. However, the drawbacks of these therapies include the acquisition of resistance and high medical costs. As an alternative approach, some scientists are already thinking of attacking the Achilles' heel of cancer. As foreseen from the presence of ferroptosis, cancers in general accumulate iron for proliferation, which may allow the abundance of catalytic iron in the cytoplasm. Indeed, hydrogen peroxide is not the only molecule to induce the Fenton reaction. Ascorbate (vitamin C) and L-cysteine as reducing agents can also initiate the Fenton reaction in the presence of catalytic Fe(III) (Toyokuni and Sagripanti, 1996). In light of this, high-dose ascorbate therapy is being tested in a clinical trial with a standard regimen of chemotherapy because ascorbate has long been proven to be a safe drug in humans (Welsh et al., 2013).

## Epilog

Iron and sulfur are essential for life despite their presence in small amounts. Excessive iron cause oxidative damage in the genome, which can be a basis of somatic mutational evolution in search of resistance against oxidative stress and cellular proliferation. Apparently, iron and thiol compounds are antagonistic toward oxidative stress, but even thiol compounds can be our foes in cancer. Therefore, it is difficult to conclude the role of iron and thiol compounds as friends or foes, which depends on the quantity/distribution and induction/flexibility, respectively. Avoiding further mutation would be the most helpful strategy for cancer prevention, and myriad of efforts are being made to sort out the weaknesses of cancer cells.

### Conflict of interest statement

The author declares that the research was conducted in the absence of any commercial or financial relationships that could be construed as a potential conflict of interest.
